# Patterns of Suicide in Kuwait: a retrospective descriptive study from 2003–2009

**DOI:** 10.1186/s12889-015-1862-7

**Published:** 2015-06-04

**Authors:** Salah Al-Waheeb, Nadia Al-Kandary

**Affiliations:** Faculty of Medicine, Kuwait University, 72, 71661 Shamiyah, Kuwait; General Department of Criminal Evidence, Ministry of Interior, Farwaniyah, Kuwait

## Abstract

**Background:**

Prior to the invasion of Kuwait by Iraq in 1990, suicides were almost unheard of in Kuwait. However, there has been a notable increase in the referrals of suicide cases to the forensic authorities since then. A review of suicide cases was performed to investigate the demographics of this phenomenon and the suicide modalities used and to uncover issues that can be addressed by the region's government.

**Methods:**

The sole source of data was the general department of criminal evidence (GDCE), where cases are referred by police authorities and by hospital investigators from the entire country. All cases signed out by forensic investigators as “suicide” during the time period 2003–2009 were retrieved. A full review of the data from the case files was made. This included demographic data, scene examination, radiographic investigations, autopsies with histo-pathological examination findings and toxicological screening results in each case.

**Results:**

A total of 347 cases were retrieved and studied. Hanging was found to be the most common suicide modality used by subjects (60 %). Non-citizens constituted 87 % of cases, and no significant difference was found between married and single subjects or between Muslims and non-Muslims. Regions that were more populated with an expatriate labour force had the highest suicide prevalence.

**Conclusion:**

The government of Kuwait needs to investigate the dire conditions in which some expatriates live and to improve their situation. More control over the dispensing of certain medications needs to be enforced. Finally, strict firearm control could help reduce the suicide rates in Kuwait.

## Background

As mandated by law in the State of Kuwait, the Office of the Medical Examiner is responsible for investigating the death of any person that occurred as a result of criminal violence, neglect, casualty, suicide, or sudden death, regardless of whether the individual was in apparent health, unattended by a physician, confined to a public institution other than a hospital, infirmary or nursing home, or was in any suspicious or unusual circumstances [1]. Complete forensic examinations are performed; these routinely include an initial scene investigation, external and internal post-mortem examinations, and toxicological and microscopic histologic studies. All findings from these examinations are reported to the criminal evidence sector in the Forensic Medicine Department (FMD) of the GDCE. The authors noted an increase in the incidence of suicide as reported by the media and as observed from the increasing number of referrals to the GDCE. This study aimed to investigate the patterns of suicide in Kuwait, one of the Arabian Gulf States, which incorporates a wide variety of multi-ethnic communities. The incidence of suicide was calculated and the effects of several demographic factors on the different modalities of suicide were studied. Moreover, the rate of suicide in some countries in the Middle East region was observed and compared to corresponding rates in other parts of the world. The ultimate goal is to demonstrate that there are public health concerns that have not been addressed by the region’s governments due to the poor ability to account for un-natural deaths in general and suicide deaths in particular in the Middle East.

## Methods

All study data were obtained from the FMD, an affiliate of the GDCE, where all medico-legal cases, including suspected cases of suicide, are referred by police authorities and hospital investigators. All case files signed out as “suicide” from the period 2003 to 2009 were retrieved, and a full review of the data was made. These data included demographic data, scene examinations, radiographic investigations and complete autopsy studies, which were performed by forensic medical examiners and entailed histo-pathological examinations and toxicological screenings to identify each cause of death.

This retrospective descriptive study included all suicides in all six Governorates of Kuwait (Ahmady, Jahra, Hawally, Mubarak Al-Kabeer, Farwaniya and Al-Asema, the latter also known as “The Capital”).

The manner of death was classified as suicide according to “A Guide for Manner of Death Classification” issued by the National Association of Medical Examiners [2]. This guide states that “Suicide results from an injury or poisoning due to an intentional, self-inflicted act, committed to inflict self-harm or cause the death of oneself.”

The regional ethical review board of the General Department of Criminal Investigations in the Ministry of Interior (MOI) of Kuwait and the Ethics Committee in the School of Forensic and Investigative Sciences at the University of Central Lancashire approved all the procedures used, provided that the confidentiality of each case was maintained.

The data were analysed with SPSS 17.0 software (Chicago, USA). Statistical analyses involved computation of descriptive statistics for all demographic factors, such as age (divided into ten-year increments: 10–19, …. up to 50–59 and 60+), gender, marital status and religion. Descriptive statistics were used to show the percentage of reported suicides in different categories according to each year. A chi square test was used to determine the association between qualitative variables. A value of p < 0.05 was considered significant.

## Results

A total of 347 suicides were reported in the GDCE during the study period.

Table 1 presents a general overview of the frequency of different demographic factors and suicide mechanisms among the total number of suicides. Table 2 presents a more detailed frequency of different demographic variables within each suicide mechanism. Important findings will be highlighted in this section.

**Table 1:** Characteristics of Kuwaiti Suicide Decedents, 2003–2009

Number (%) of total number of suicides	Category
13(4)	Jump from Height
18(5)	Firearm
49(14)	Poisoning
58(17)	Sharp Objects
209(60)	Hanging
242(70)	Male
105(30)	Female
44(13)	Kuwaiti
303(87)	Non-Kuwaiti
169(49)	Married
178(51)	Non-Married
171(49)	Muslim
176(51)	Non-Muslim
54(16)	Jahra
137(39)	Farwaniyah
36(10)	Hawally
68(20)	Ahmady
14(4)	Mubarak AL-Kabeer
38(11)	Capital

**Table 2:** Number (%) of suicides by method as they relate to gender, nationality, marital status and Governorate

	Jump from height	Firearm	Sharp objects	Poisoning	Hanging
Male	4(31)	18(100)	52(90)	32(65)	136(65)
Female	9(69)	0(0)	6(10)	17(35)	73(35)
Kuwaiti	0(0)	12(67)	10(17)	3(6)	19(10)
Non-Kuwaiti	13(100)	6(33)	48(83)	46(94)	190(90)
Married	2(15)	11(61)	34(59)	28(57)	94(45)
Non- Married	11(85)	7(39)	24(41)	21(43)	115(55)
Muslim	10(77)	16(89)	32(55)	17(35)	96(46)
Non-Muslim	3(23)	2(11)	26(45)	32(65)	113(54)
Jahra	1(8)	2(11)	10(17)	7(14)	34(16)
Farwaniya	3(23)	8(44)	18(31)	19(39)	89(43)
Hawally	5(39)	0(0)	7(12)	5(10)	19(9)
Ahmady	2(15)	5(28)	13(24)	9(18)	39(19)
Mubarak Al-Kabeer	0(0)	0(0)	1(2)	3(6)	10(5)
Capital	2(15)	3(17)	9(16)	6(12)	18(9)

Significantly more people died from hanging compared to other suicide categories (p < 0.05), while jumping from a height was the least utilized method of suicide and was exclusively found in non-Kuwaiti cases. More males died from suicide compared to females in all suicidal method categories (p < 0.05) except for jumping from a height. Moreover, all methods of suicide were utilized at a higher rate in the non-Kuwaiti population (p < 0.05) with the exception of suicide by firearm. There was no statistically significant difference between married and non-married groups or between Muslim and non-Muslim subjects.

The highest number of suicides occurred in the Farwaniya region, and the lowest number occurred in the Mubarak Al-Kabeer region (p < 0.05).

Table 3 shows the relationship between age groups and the different suicide categories. All suicides were between the ages of 17 and 62. The 30–39 age group accounted for most of the cases of suicide by sharp objects and poisoning (p < 0.001). The fewest cases were recorded in the over 60 age group.

**Table 3:** Number (%) of suicides by age group and method

	Jump from height	Firearm	Sharp objects	Poisoning	Hanging
10-19	0(0)	1(6)	2(3)	0(0)	4(2)
20-29	7(54)	4(22)	14(24)	19(39)	82(39)
30-39	5(38)	7(39)	29(50)	24(49)	85(41)
40-49	0(0)	3(17)	7(12)	5(10)	28(13)
50-59	1(8)	3(17)	4(7)	1(2)	9(4)
>60	0(0)	0(0)	2(3)	0(0)	1(0.5)
Total	13	18	58	49	209

Suicide by hanging was highest among the 20–29 and 30–39 age groups (p < 0.001). The over 60 and under 19 age groups had the lowest number of suicides by hanging.

There was a significant difference (p < 0.001) in the number of suicide cases due to injuries sustained by jumping from a height in the 20–29 and 30–39 age groups compared with the other age groups.

The highest number of suicides by firearm injuries occurred in the 30–39 age group. In contrast, no case of suicide by firearm was reported in the over 60 age group.

Table 4 shows a list of suicide rates in 2002 for Middle Eastern Countries from the World Health Organization (WHO) statistics database (WHO). The data show that in the year 2002, the lowest suicide rate was recorded in Syria, and the highest rate within the Gulf Countries, 5.4, was recorded in Bahrain. The highest rate in the Middle East was in Israel. Kuwait had a suicide rate of 3.2, which is considered to be high compared to the neighbouring and more populated nation of Saudi Arabia, which has a rate of 1.2.

**Table 4:** Comparison of suicide rates in Middle Eastern Countries (per 100,000) by country and gender for 2002

Serial no.	Country	Males	Females	Total
1	ISRAEL	10.5	2.6	13.1
2	BAHRAIN	4.9	0.5	5.4
3	EGYPT	0.1	0.0	0.1
4	IRAN	0.3	0.1	0.4
5	JORDAN	0.0	0.0	0
6	KUWAIT	1.6	1.6	3.2
7	SAUDI ARABIA	2.6	1.4	1.2
8	QATAR	0.9	0.4	0.5
9	JORDAN	0.3	0.8	1.1
10	SYRIA	0.2	0	0.2
11	YEMEN	1.2	0.4	1.6

## Discussion

This study was designed to investigate the patterns of suicide in the State of Kuwait. It is the first of its type to study the different demographic parameters (age, gender, religion, nationality and Governorate) in the reported suicides in this country. It was of paramount importance to perform the necessary analyses to determine the basis of the suicide problem in Kuwait. This will subsequently help the Kuwaiti Government tackle the problem and reduce the number of suicides in the country.

The results presented in this study highlight a number of interesting details, which will be discussed in the following account. We will also briefly discuss the rates of suicide in other Middle Eastern Countries, mainly for comparison with our study, as many of these countries share similar social and cultural parameters with Kuwait.

Suicide is one of the leading causes of death in the world, and suicidal attempts constitute a major public health concern [3]. The total number of 347 cases in our study was small compared to the absolute numbers reported from many other countries around the globe. For a country such as Kuwait with a population of approximately 1.5 million in 2003 and 2 million in 2009, this number is quite high.

Hanging was the leading method of suicide, while jumping from a height was the least common method. A possible explanation is that jumping from a height constitutes a horrible experience for the person committing suicide. Moreover, it may be viewed and noticed by others. On the other hand, suicide by hanging has some privacy associated with it, although the mechanism of death is complex [4].

Males outnumbered females in all methods of suicide except in jumping from a height, for which the percentage of females was greater. Saudi Arabia shares a border with Kuwait, and the two countries share an almost identical culture. In Saudi Arabia, 82 % of suicides were among males [5]. All victims of firearm injuries in Saudi Arabia were males. The most plausible explanation for this observation is that gun ownership is limited to men by regulations of the country.

The most common method of suicide in our study and in similar studies in the Middle East is hanging [6]. In contrast, in a Scottish study, poisoning was the most preferred method of suicide [7]. Rates of suicide in most countries in the world, including Denmark, are higher among males, which is consistent with our results [8, 9]. China is an important exception, with a very high suicide rate among females, especially young women in rural areas [10]. However, there has been a recent trend of reduced female and increased male suicide in China [11, 12].

The results of this study have clearly shown that all categories of suicide occurred more frequently among the non-Kuwaiti population, except for firearm injuries. Jumping from a height was reported exclusively among non-Kuwaiti suicide cases. Most of these victims were housemaids who could not run away from the ill treatment of their employers. Similar results were reported in Bahrain, another neighbouring gulf country with similar customs and traditions to Kuwait. The mean suicide rate was 0.6 per 100,000 for Bahraini nationals and 12.6 per 100,000 for expatriates. Indian immigrants in Bahrain demonstrated the highest suicide rate [6]. Similarly, in Saudi Arabia, more expatriates (65.4 %) committed suicide than Saudi citizens (36.4 %) [13].

No significant difference in suicide rates was observed between married and single subjects. However, other studies comparing socio-demographic characteristics revealed that a higher prevalence of suicide was associated with female gender, lower education, poor financial perception and single marital status [14, 15].

There is limited data in the literature on the relationship between marital status and suicidal behaviour in the Middle East [6]. A recent study from Japan concluded that single people committed suicide more often than married people [16]. New reports in 2010 published by the Office for National Statistics in the United Kingdom (UK) indicated that suicide rates among single and divorced subjects were approximately three times higher than corresponding rates in married subjects of both genders between 2000 and 2009 [17]. Their results showed that 30.8 single men per 100,000 died by committing suicide in the UK, compared to 10.6 per 100,000 married men. In the same period, 10.3 single women per 100,000 succumbed to suicide compared to 3.6 per 100,000 married women. The authors suggested that single people of either gender are more vulnerable to depression than married people.

Farwania Governorate ranked first in suicide prevalence followed by Ahmady, Jahra, the Capital, Hawally and Mubarak Al-Kabeer. The Farwania and Ahmady Governorates are heavily populated with expatriate labourers who work at factories and cleaning companies. These Governorates have many buildings where apartments are sublet by tenants in order to earn more money or cover their own expenses. These areas are therefore overcrowded and difficult to monitor by authorities. As a result, suicides are higher in these Governorates. Data provided by the MOI in March 2010 revealed that the Farwania and Ahmady Governorates have high crime rates [18]. Many individuals who live in these Governorates possibly retaliate by committing crimes or become part of a criminal gang to gain extra income. It is now well known that these depressed areas in Kuwait are a true fortress for many organized crimes, and as a result, many people are stressed and under severe pressure due to gang crimes. Depression and stress are forerunners of suicide.

Significantly more people in our study committed suicide in their prime and productive years of life compared to other periods. Approximately 71 % were between the ages of 20 and 39. The extreme age categories were the least vulnerable to suicide, with a prevalence of 5.7 % in the under 19 group and 2.9 % in those over 60. Similar results were found in India, where studies have highlighted that the most common age group of suicide victims was 21–30 [14, 15]. In contrast, in the United States of America (USA), a study in 2009 showed that suicide was the third leading cause of death for young people between the ages of 15 to 24 [19, 20].

Traditionally, poison (poisoning) has been one of the most commonly used substances for suicide and homicide. In our study, suicide due to poisoning was the third most common suicide modality. A study conducted in Egypt revealed that female adolescents are at a higher risk of self-poisoning [21]. Deliberate self‐harm is a major problem in the developing world; widespread availability and easy access to poisons make it easier for the vulnerable to commit suicide [22]. This situation is quite often found in cases of drug overdose, which is another method of committing suicide [23]. A study was conducted in an addiction treatment centre in a psychiatric hospital in Kuwait to review cases of poisoning in order to identify the main poisons used for self‐harm in this region [24]. The results showed that pesticides were the most prevalent type used, followed by paracetamol (acetaminophen). Both of these products are readily available in any grocery store.

In 2009, the government of Kuwait reported that most cases of attempted suicide were by ingestion of pesticides or paracetamol (acetaminophen) [25]. These drugs have become the most popular method of self‐harm due to their ready availability in residential areas, especially among people from the Bangladeshi community who live in Kuwait. In India, medicines such as benzodiazepines and antidepressants were those most commonly used by victims of self-poisoning [14, 15]. The previous data provide strong evidence to support the restriction of some drugs that are easily available over the counter in shops and in supermarkets to combat the blatant abuse that can lead to suicide [26, 27].

Suicide by firearm injuries was uncommon in our study. Guns are one of the most effective means for committing suicide and are a far more prevalent suicide method than drug overdose, strangulation or cutting oneself in the USA [28]. Given the high rate of gun ownership in the USA, it is logical to expect a direct link between increased access to guns and a high incidence of suicide. However, international data show that higher levels of gun ownership do not always correlate with higher levels of suicide [29]. For example, personal gun ownership is more prevalent in Switzerland than in the USA, but suicide rates are higher in the USA [30]. The actual chain of events leading to selection of a firearm as a means of death is unclear. In fact, there are some mediating factors that contribute to firearm suicide. People consider suicide by firearm as an honourable and quick way to eliminate personal troubles and disappointments. Easy access to dangerous weapons is also an important risk factor. Many Kuwaitis have guns in their possession as a result of the gulf war in 1990, and many campaigns are spearheaded by the MOI to collect weapons that are in the possession of residents and citizens without a valid permit.

Table 4 compares suicide rates in the Arabian Gulf and selected Arab and Middle eastern countries in the year 2002 [31]. Kuwait had one of the highest rates in the region. The suicide rate in Kuwait has increased from 3.2/100,000 in 2002 to 6.1/100,000 in 2010 [31], while the rate fluctuated between these two values throughout the period of the study. The literature has limited reports on suicide in the Middle East. However, the 2002 rates according to the WHO website (http://apps.who.int/en/) are similar to the rates observed during the study period (2003–2009). Reports of the suicide rate in Bahrain in 1988 show similar values to that in Kuwait (5.4/100,000) [31] and have increased in 2012 to reach 8.1, according to the WHO website. Most Arabic families of suicide victims would never admit the actual cause of death to anyone and always state that their son or daughter died suddenly. Moreover, due to the negative views that the general population has against autopsies, many suicides probably do not reach the forensic office, as families will try and bury their loved ones quickly and are compassionately given death certificates from authorities without the word ‘suicide’ written anywhere. Saudi Arabia views suicide as a disgraceful and shameful act more than any other Arab or Muslim country [32]. Similar results have been found in the United Arab Emirates, in which the annual rate of suicide in Dubai between 1992 and 2000 was approximately 6.2/100,000 [33, 34]. We found no significant difference in suicide incidence between Muslims and non-Muslims, despite the fact that suicide is prohibited in Islam and is regarded as a sinful act similar to homicide. It appears that the stresses and probable depression that suicide victims feel prior to their demise supersede any religious teachings.

## Conclusion

The rate of suicide in Kuwait is higher than that in several neighbouring countries, and we feel this rate is an underestimate due to religious and cultural reasons that have been addressed previously.

The dire living conditions in the heavily populated Governorates in Kuwait need to be assessed by government officials to decrease the stresses that the expatriate workforce are exposed to.

Because jumping from a height was most commonly reported among female house workers, this demographic group needs to be supported by the government of Kuwait. This support could be carried out by implementing rules and regulations that protect home caregivers against the abuse of their employers.

Although not a common form of suicide, deaths by firearm can be controlled by enforcing stringent rules preventing people from owning rifles and handguns.

## Abbreviations

FMD: Forensic Medicine Department
GDCE: General department of criminal evidence
MOI: Ministry of interior

## References

[1] Al-Dousery, F.Ministry of interior. Kuwait. Criminal Evidence between theory and practice. PhD Thesis.1994, P: 34–37.

[2] Hanzlick, R., Hunsaker, J. and Davis, G. “A Guide For Manner of Death. Classification,” First Edition, National Association of Medical Examiners. 2002, pp 46–59

[3] Omar HA, Merrick J (2013). The young and suicide. Int J Adolesc Med Health.

[4] Elfawal M, Awad O (1997). Firearm Fatalities in Eastern Saudi Arabia: Impact of Culture and Legislation. Am J Forensic Med Pathol.

[5] Al Madni OM, Kharoshah MA, Zaki MK, Ghaleb SS (2010). Hanging deaths in Dammam, Kingdom of Saudi Arabia. J Forensic Leg Med.

[6] Al-Ansary A, Razaq M, Ali A (2007). Suicide in Bahrain in the last decade. Forensic Journal of Psychiatry.

[7] Arun M, Palimar V, Kumar J, Menezes RG (2010). Unusual methods of suicide: complexities in investigation. Med Sci Law.

[8] Spicer R, Miller T (2000). Suicide acts in 8 states: incidence and case fatality rates by demographics and method. Am J Public Health.

[9] Yong E (2009). Attendance at religious services, but not religious devotion, predicts support for suicide attacks. J Psychol Med.

[10] Chui M, Tilly L, Moulton R, Chui D (2002). Suicidal stab wound with a butter knife. Can Med Assoc J.

[11] Xiang YT, Yu X, Sartorius N, Ungvari GS, Chiu HF (2012). Mental health in China: challenges and progress. Lancet.

[12] Sun J, Guo X, Zhang J, Jia C, Xu A. Suicide rates in Shandong, China, 1991–2010: Rapid decrease in rural rates and steady increase in male–female ratio. J Affect Disord. 2012 Oct 12. doi: 10.1016/j.jad.2012.09.020. 10.1016/j.jad.2012.09.020 23068020

[13] Elfawal M (1999). Cultural Influence on the Incidence and Choice of Method of Suicide in Saudi Arabia. Am J Forensic Med Pathol.

[14] Vijayakumar L (2010). Indian research on suicide. Indian J Psychiatry.

[15] Radhakrishnan R, Andrade C (2012). Suicide: An Indian perspective. Indian J Psychiatry.

[16] Ishii N, Terao T, Araki Y, Kohno K, Mizokami Y, Arasaki M, Iwata N. Risk factors for suicide in Japan: A model of predicting suicide in 2008 by risk factors of 2007. J Affect Disord. 2012 Dec 19 10.1016/j.jad.2012.11.038 23261137

[17] Office for National Statistics Report. United Kingdom: Government of the United Kingdom; 2010.

[18] Ministry of Interior Report. Kuwait: Government of the State of Kuwait; 2011.

[19] Miller M, Hemenway D (2008). Guns and Suicide in the United States. N Engl J Med.

[20] Denney J, Rogers R, Krueger P, Wadsworth T (2009). Adult Suicide Mortality in the United States: Marital Status, Family Size, Socioeconomic Status, and Differences by Sex. Soc Sci Q.

[21] Shreed S, Tawfik N, Mohammed N, Elmahdi M (2011). Toxic agents used for parasuicide in Damietta Governorate, Egypt. Journal of Middle Eastern Current Psychiatry.

[22] Linehan M, Comtois K, Murray A, Brown M, Gallop R, Heard H, Lindenboim N (2006). Two-year randomized controlled trial and follow-up of dialectical behavior therapy vs. therapy by experts for suicidal behaviors and borderline personality disorder.. J Genet Psychol.

[23] Hunt I, Kapur N, Webb R, Robinson J, Burn R, Turnbull P, Shaw J, Appleby L (2007). Suicide in current psychiatric in-patients: a case–control study. J Psychol Med.

[24] Fido AA, Al MA (1989). Consultation liaison psychiatry in a Kuwaiti general hospital. Int J Soc Psychiatry.

[25] Toxicological Division report. Ministry of Health. Kuwait: Government of the State of Kuwait; 2009

[26] Hawton K (2002). United Kingdom legislation on pack sizes of analgesics: background, rationale, and effects on suicide and deliberate self-harm. Suicide Life Threat Behav.

[27] Hawton K, Ware C, Mistry H, Hewitt J, Kingsbury S, Roberts D, Weitzel H (1995). Why patients choose paracetamol for self poisoning and their knowledge of its dangers. Br Med J.

[28] Miller M, Azrael D, Hemenway D (2002). Household Firearm Ownership and Suicide Rates in the United States. Epidemiology.

[29] Wilson J (2010). Localized homicide patterns and prevention strategies : a comparison of five project: Safe neighborhood. J For Sci.

[30] Tewksbury R, Geetha S, Ronald M (2010). Factors Related to Suicide Via Firearms and Hanging and Leaving of Suicide Notes. International Journal of Men's Health.

[31] World Health Organization.Un-Natural deaths, country, regional and global estimates. Geneva: WHO; 2006, 2(993):129–146

[32] Al-Barraq A, Farahat F (2011). Pattern and determinants of poisoning in a teaching hospital in Riyadh Saudi Arabia. Saudi Arabia Pharmaceutical Journal.

[33] Benomran A, Hassan A (2007). Masking and bondage in suicidal hanging. J Forensic Legal Med.

[34] Koronfel A (2002). Suicide in Dubai. J Clin Forensic Med.

